# Skin grafting treatment of adolescent lower limb avulsion injury

**DOI:** 10.3389/fsurg.2022.953038

**Published:** 2022-09-15

**Authors:** Liu Yang, Jiachao Guo, Jinpeng He, Jingfan Shao

**Affiliations:** Department of Pediatric Surgery, Tongji Hospital, Tongji Medical College, Huazhong University of Science and Technology, Wuhan, China

**Keywords:** adolescent, avulsion, skin graft, NPWT, SCAR

## Abstract

**Background:**

Under the influence of various factors, the number of lower extremity avulsion injuries in adolescents is increasing year by year. The main modality of treatment is skin grafting. There are many types of skin grafting. Although many studies on skin grafting after avulsion injuries have been published in the past few decades, there are differences in the treatment options for adolescents with post avulsion injuries.

**Main body:**

Thorough debridement and appropriate skin grafts are essential for the surgical management of avulsion injuries for optimal prognosis. In the acquisition of grafts, progress has been made in equipment for how to obtain different depths of skin. The severity of the avulsion injury varies among patients on admission, and therefore the manner and type of skin grafting will vary. Especially in adolescents, graft survival and functional recovery are of great concern to both patients and physicians. Therefore, many efforts have been made to improve survival rate and activity.

**Conclusion:**

This review summarizes the principles of treatment of avulsion injuries, the historical development of skin grafts, and the selection of skin grafts, hoping to be helpful for future research.

## Introduction

Large-scale avulsion injuries in adolescents are rare, but due to the rapid construction and development of cities, traffic accidents, heavy object impacts, and large-scale mechanical trauma have increased year by year, and the number of patients with these injuries has increased rapidly (1, 2). In China, due to the opening of the three-child policy, the proportion of adolescents in the population has greatly increased, which has also led to an increase in the incidence of such patients. Adolescents are different from adults in that they have very poor awareness of self-protection and cannot protect themselves from accidental injuries. Therefore, there is a high incidence of lower extremity avulsion injuries in adolescents. The skin structure of adolescents is thinner than that of adults, and the strength of the skin structure and subcutaneous tissue is weak, and it is very easy to have skin avulsion when suffering from trauma. In addition, adolescents have better bone flexibility, and avulsion of the skin is often seen in some traumas without fractures, which is different from adults. The absence of fractures in adolescent results in incomplete release of energy, making them more prone to avulsion injuries (3). The avulsion injury of the lower extremity is different from that of the upper extremity. The lower extremity has a large range of motion and also bears most of the weight of the human body. Increased hydrostatic pressure, predisposing to seroma/hematoma (2). Since adolescents are still in the stage of growth and development, severe scarring occurs in the later stages of lower extremity avulsion injuries in adolescents, which can lead to severe functional deficits (4, 5).

Treatment of large avulsion injuries of the lower extremity in children is a great challenge for surgeons. Avulsion injuries are often accompanied by other serious concurrent or underlying injuries. These co-injury and underlying injuries not only require attention, but also complicate the management of avulsion injuries (6). After processing the combined injury, prepare the wound bed according to the TIME framework (Tissue, Infection/inflammation, Moisture balance, Epithelial edge advancement) (5, 7, 8). The use of grafts is an irreplaceable part of the treatment of avulsion injuries. The advent of grafts offers hope for patients with large skin defects. Grafts are divided into autologous skin grafts and artificial grafts. At present, since the invention of artificial dermis in 1980, it has been widely known for its simplicity and abundant availability, but the need for skin grafting in the second stage operation also limits its widespread use to a certain extent (9–11).

Autologous skin grafts have always been the main treatment for wound reconstruction, and can be divided into split-thickness skin grafts(STSGs), full-thickness skin grafts(FTSGs), and epidermal grafts(EGs) based solely on the composition of the skin (12, 13). Its use requires the surgeon to judge the severity of the wound and choose the appropriate treatment. In this review, we searched PubMed, China National Knowledge Infrastructure(CNKI), and Ovid and reviewed the titles/abstracts of studies on avulsions and skin grafts from 1992 to the present, with no language or date restrictions on the use of “avulsion” and “avulsion” skin grafts” (see appendix for search strategy). We have elaborated various transplantation methods in detail, hoping to provide guidance for clinicians, Reduce adverse effects on patients due to inappropriate treatment modalities, allowing surgeons to make better clinical judgments. In addition, with the development of new biomaterials and surgical methods, better treatment options for avulsion injuries in adolescents may be provided in the future.

## Definition and evaluation of lower extremity avulsion injuries

Extensive avulsions are usually caused by rolling over a wheel or tire in a mechanical accident. It refers to the separation of the skin and subcutaneous fat layer from the underlying tissues (fascia, muscle, and bone) by abruptly strong shear forces, and the affected skin is greater than one-quarter of the total area of the lower extremity (14). Improper handling can lead to necrosis of the avulsion flap, infection, and even the risk of amputation (14, 15). Avulsion injuries of the lower extremities are mostly traffic accidents and may be combined with multiple injuries throughout the body (16). Therefore, it is often manifested not only in wound bleeding, pain, and extensive skin avulsion, but also in complications such as hemorrhagic shock, organ rupture and bleeding, and pelvic fractures. Compared with adults, adolescents have weaker verbal skills and cannot describe their feelings correctly. And the condition of young people changes more rapidly, and may further deteriorate at any time. Therefore, surgeons need to assess the patient's vital signs in a timely manner, and in addition to immediate surgery, they also need to carry out corresponding treatment measures, such as simple wound dressing, blood transfusion, and appropriate fluid management (5, 17). Adolescents do well in the short term due to its rapid healing ability. Scar contracture may occur with the growth and development of adolescents, so that patients may be troubled by limited joint mobility, joint deformity, and aesthetic appearance in the future.

According to the magnitude of the impact energy, avulsion injuries can be divided into 3 types: (1) Simple avulsion injuries, divided into circumferential and non-circumferential avulsions, deep soft tissues, muscles and bones are not significantly damaged; (2) Avulsion injuries in which the injury reaches deep soft tissues, divided into circumferential and non-circumferential avulsions, where the muscles are partially involved, but the long bones are not fractured; (3) Avulsion injuries of cumulative long bones, this high-energy injury has severe skin and muscle contusion, and the risk of infection high. Types 1 and 2 have significantly better prognosis than type 3 (14).

## Principles of treatment of avulsion injuries

The treatment of avulsion injuries first requires preparation of the wound bed. The treatment measures corresponding to the TIME framework are thorough debridement, antibacterial disinfection, humidity regulation and improvement of microcirculation (8, 18, 19). Avulsion injuries are typically heavily contaminated open wounds whose initial management requires extensive irrigation and thorough debridement and removal of all inactive and abnormally nonfunctional tissue (20–22). However, the choice of irrigation pressure and irrigation solution is controversial. Bhandari M et al. conducted a study on wound irrigation and showed that high pressure can better remove foreign bodies and bacteria, which is suitable for foreign bodies that are difficult to remove; low pressure can avoid tissue It is suitable for wounds with no foreign body or easy to clean (16, 23). Skin defects caused by avulsion injuries cannot be solved by simple skin closure. The skin defects formed after excision can be covered with a vacuum-assisted closure system first, followed by wound conditioning and skin transplantation (5, 15, 24). Treatment within 8 h of injury is considered to be the golden treatment time, and delayed admission may increase the risk of postoperative infection, reduce flap survival rate and treatment efficacy (1). In the treatment of avulsion wounds, antibiotics are generally used to prevent wound infection. Antibiotic guidelines recommend, extremity wounds (cefazolin, 2gIV q6–8 h, 1–3 days) (25). In particular, microbial testing is necessary. For large-area avulsion injuries/deep tissue injuries, Gram-positive Rough bacillus smear testing is required to check for gas gangrene. If it is positive, the negative pressure suction technique should be used with caution, the wound should be opened and washed repeatedly with hydrogen peroxide. Specimens should also be collected and sent for bacterial culture to provide evidence-based evidence for subsequent systemic intravenous antibiotics and topical medication. It is necessary to inject tetanus immune globulin at the same time. The avulsion injury is a deep wound, and Clostridium tetani is easy to multiply (26). Moisture balance in the wound bed aids epithelial cell migration, and dressing selection is important, including protease-modified dressings as well as superabsorbent dressings and skin barrier creams (27). In addition, fluid management is also an indispensable point. Imbalanced fluid balance, decreased blood volume, insufficient perfusion of important organs and insufficient oxygen supply to tissues will seriously affect the normal function of the body.

After the initial treatment of the wound, the main approach to the treatment of large skin defects is to choose graft coverage. The conventional graft of choice is autologous skin. When the patient's skin nutritional status is poor, or even suffering from diabetes, the healing of the donor site will be troubled. At this time, new artificial substitutes can be used (28). The long-term results after skin grafting are always suboptimal. Severe scars develop gradually over the years after the trauma, and the scars will develop secondary contractures. Adolescents are more likely to develop limb contractures than adults because scars do not grow proportionally with the body (29). Adolescents with scar-related problems require multiple soft-tissue reconstruction surgeries, long-term follow-up by doctors, and close attention to adolescents' psychological problems.

## Skin grafting

### Molecular mechanism of skin grafting

Skin graft healing can be divided into three phases: plasma absorption phase, contact phase, and revascularization phase (30). The plasma absorption phase is when the transplanted tissue absorbs the tissue fluid secreted by the wound. The contact period is when anastomosis occurs between the blood vessels of the donor skin and the recipient site in the dermis (31). The keratinocyte activation phase, on the other hand, appeared only in epidermal transplantation, possibly due to the marked expression of Ki67 (a marker of cell proliferation) and the *β*1 integrin subunit (a putative marker of keratinocyte stem cells) (32). The revascularization stage is the stage of vascular proliferation, and vascular endothelial growth factor (VEGF) is one of the regulatory factors. The increase of capillary permeability leads to the increase of extravascular tissue fluid. VEGF-mediated neovascularization (29). The exact mechanism is still unclear. However, blood vessels are not involved in epidermal transplantation, so this stage is skipped in epidermal transplantation and epithelization is directly carried out to achieve the purpose of repair (33–35).

### Development of skin grafts

Skin grafting is when skin tissue is detached from one site and transplanted to another site, with the transferred skin merging into the recipient site (36, 37). Skin grafting is the most critical part of treatment. If large-scale skin defects are not treated effectively, infection and a series of complications due to prolonged bed rest will occur. In addition to the treatment of avulsion injuries, skin grafts can also be used for skin cancer surgery, leg ulcers caused by diabetes, etc. (38, 39). Skin grafts for repairing nasal injuries were first described by Indian surgeons in 2,500 BC (40). In 1872, Ollier de Lyon described the first STSG technique (39). The STSG technique depends on the depth of graft acquisition in the donor site, so three types of instruments have been designed, including skin grafting knives, drum dermatomes, and electric dermatomes, to speed up the application of STSG (41–43). In 1964, J.C. Tanner et al. developed mesh skin grafts based on STSG (44). FTSG was first described in 1875 by Wolfe et al. (45). FSTG is more prone to necrosis than STSG, but since FTSG is composed of intact dermis and epidermis, scar shrinkage is less (46).

The epidermal blister technique was first described in 1964 by Kiistala et al. (47). Their technique proposes to harvest the epidermal layer of the skin by forming water-absorbing vesicles, providing autologous keratinocytes for transplantation. FTSG and STSG usually require hospitalization, and epidermal grafting can be done as a day surgery. And since there is no damage to the dermis of the donor site, the donor site will not leave scars (12). In 1981, since John Burke et al. invented the artificial skin dermis called Integra consisting of silicon epidermis and porous collagen-chondroitin (48). Various types of skin substitutes have been put into use, epidermal substitutes such as Cultured epidermal autograft (CEA), Epicel, Cell Spray, dermal substitutes such as Matriderm, Biobrane, Pelnac, composite skin substitutes such as Apligraft, StrataGraft, OrCel etc. These become alternatives in the field of reconstructive surgery (10, 49–52). Among the many advantages of all types of skin substitutes, the most prominent are simplicity, immediacy, and plentiful availability (53, 54). However, its use in large skin defects is limited due to its high cost and need for secondary surgery (Table 1).

**Table 1:** Different types of skin grafts.

Types of skin grafts	Application	Advantages	Disadvantages	Highlights
FTSG	Repair defects on the nasal tip, dorsum, ala, and sidewall, as well as on the lower eyelid and ear Palm, sole, ankle, knee	Less prone to scar contractures	The damage to the donor area is large, and it is prone to necrosis after transplantation	It is recommended to take the skin from the groin, a lot can be taken, and it can effectively reduce the scar contracture. In addition, the scar is hidden and beautiful. It can also be taken repeatedly, the scar in the receptor area is smaller, and the color is closer to normal skin.
STSG	Skin Defect After Resection of Invasive Tumor Diabetic foot or leg ulcers Areas with low activity needs, such as calves, thighs	Better nutrient diffusion Easy to survive	Prone to scarring contractures Developing hypopigmentation or hyperpigmentation	The skin extraction area is larger, the thickness of the skin slice is uniform, and it is easy to survive.
EG	White spots caused by vitiligo Acute/chronic superficial wounds	Painless No scarring at the donor site No need for anesthesia	The epidermis needs to be obtained from the own skin at the same time, and almost the same amount of donor site is required.	It is mainly aimed at the huge defect area, which can cover the largest area with the least skin, mainly relying on the crawling coverage of epidermal cells, and the skin island can effectively induce the crawling of epidermal cells. easy to survive.
Skin Substitutes	Exposed bone, exposed tendon Finger stump, diabetic non-healing wound Donor area	Repairs skin defects on its own Easy access No peeling required	The dressing cycle is slightly longer Some wounds require a second-stage autologous skin graft	Durable, can be mass-produced, reduce the need for donor tissue, and can cover large areas of wounds

### Full-Thickness skin grafts

FTSGs contains full-thickness dermis, and most surgeons use it for facial reconstruction, joint mobility, and part of the lower extremity skin defect. FTSG is less recommended for infection-type wounds, and smoking and bleeding disorders are relative contraindications to FTSG (55, 56). In facial plastic reconstruction surgery, the requirements for appearance are very high, so grafts with less actinic damage are usually selected. Such as the area behind the ear, the skin of the upper eyelid, or the inside of the arm (57). In lower extremity skin defects, full-thickness grafts are an effective reconstruction method for some defects that are difficult to close after primary closure (58). Defects larger than 1 cm in diameter require sufficient granulation tissue to form on the surface so that FTSG is not prone to necrosis (59, 60). To match the texture, color, thickness, and actinic damage of the skin at the recipient site, adjacent skin is ideal. However, when there is a large defect, it is not possible to obtain enough full-thickness grafts, and skin from other parts needs to be selected as the donor. Therefore, the selection of FTSG donor sites is very difficult. In addition, complications such as infection, hematoma/seroma, venous thrombosis, and graft necrosis may occur in lower extremity FTSG (61). Because the skin tissue obtained from the donor site includes deep dermal structures such as hair follicles and sweat glands, it can cause permanent scarring at the donor site. Scar tissue shrinks with age, leading to deformities (62). Notably, Herskovitz et al. found that a full-thickness skin column was collected using a custom-made device that could be applied directly to the wound. The donor site heals with little scarring. However, the pig model was used, and its clinical application needs further research (62). At present, there is no good solution to the damage caused to the donor site.

Pain recovery from full-thickness skin grafts in the recipient area It has long been thought that the regenerative fibers of the grafted skin grow into the graft independently of the existing Schwann sheath. That is, a healthy wound bed and a satisfactory graft, the final feeling is close to that of the surrounding skin. Napier JR observed that most FTSG were normally reinnervated around the periphery, while in the central region, the grafts were less sensitive. And Napier showed through a series of experiments that the original nerve plexus of the transplanted skin determines the scope and quality of reinnervation (63). It is generally accepted that full-thickness skin grafts have the smallest shrinkage of all autografts. Compared with STSG, the ability to resist contraction is stronger (64, 65). But A.J. Stephenson et al., the first objective measurement in humans, found that the graft shrunk by a third in the absence of infection in the wound; when infection did occur, the wound shrunk by a factor of two. This means that considering the amount of FTSG shrinkage in advance during reconstruction can reduce the effect of late grafts on function to a certain extent (66). Contractures of the graft at the joints of the lower extremities (knee, ankle and toes) can result in limited joint function and walking deformities. Transplantation in adolescents requires predicting graft growth and stretching variables. FTSG has the least shrinkage and is the most suitable type of graft for this type of site. Physical therapy and splints are also used to avoid contractures. Manju D et al. studied the contracture rate of different grafts in 174 children with finger burns and showed that the use of full-thickness skin grafts resulted in a lower rate of contracture and reoperation, especially when the palm and extended into the fingers. Graft contracture rates at lower extremity joints may be similar (46).

Immobilization is required after FTSG to improve the success rate of transplantation due to increased hydrostatic pressure during lower extremity activity and walking (58). However, numerous studies have shown that STSG of the lower extremity usually does not require immobilization (67, 68). Methods of fixation include sutures and physical compression therapy. Two suture methods are commonly used in FTSG, through suture and quilted graft. S. Struk et al. described that penetration sutures with short-term immobilization of lower extremity grafts using plaster casts or compression bandages can achieve graft completion rates as high as 93% (58). Gagik oganesyan et al. fixed the graft with penetrating suture, and the FTSG was superior to the STSG in terms of graft survival and aesthetics (69). The grafting technique using quilting has been applied in many settings, with Patterson for the hand and face, Liew S for nipple reconstruction, and Mc Gregor IA for the oral cavity (70–72). Isaac Harvey et al. used quilted full-thickness grafts for the first time to cover lower extremity defects. The risk of postoperative infection, hematoma, and seroma is greatly reduced (61).

### Split-Thickness skin grafts

**Figure 1:**
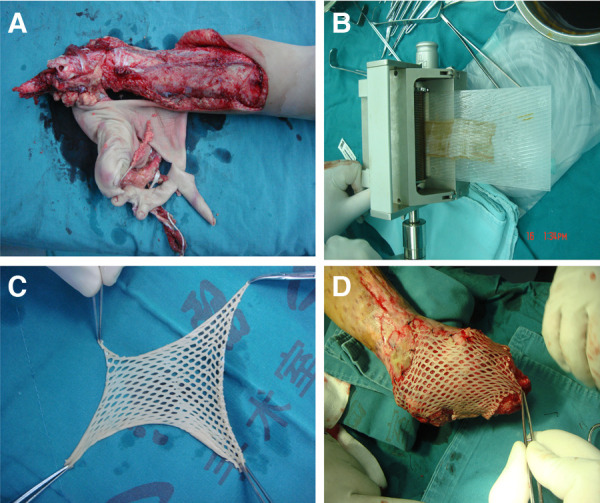
Split-thickness skin grafts meshing. (**A**) A severe juvenile lower extremity avulsion injury. (**B**) Graft meshing using a peeler. (**C**) Split-thickness skin grafts that has been meshed. (**D**) To cover the graft on the wound surface.

STSGs consists of the epidermis and a part of the dermis. The scope of application of STSG is wider than that of FTSG. STSG can not only cover thick skin grafts of different depths. It can also be used for traumatic or previously infected wounds, such as avulsion wounds, chronic leg ulcers, etc., due to the rapid osmotic transfer of nutrients from the underlying wound bed (13). However, it is not very suitable for joints, and the shear force generated by joint movement can lead to graft failure (36). Mesh grafts are full-thickness or thick-thickness skin grafts cut into staggered slits arranged in parallel. STSG is meshed and can be stretched into any shape to the greatest extent to meet the needs of irregular wounds (Figure 1). And each additional mesh incision multiplies the skin border, speeding up the healing time. It can also drain the accumulated blood in time to avoid infection. However, permanent, unsightly “fishing net” pattern scars are also caused on the grafted skin (62, 73). Avulsion injuries of lower extremities often require large-scale skin grafting, but patients often have numerous concurrent injuries, poor skin nutritional status, and it is difficult to choose a donor site. There is also the risk of pain, scarring and infection at the donor site, which is more troubling to the patient than the primary wound. STSGs are usually translucent and smooth, so it is impossible to achieve tissue texture matching (36).

**Figure 2:**
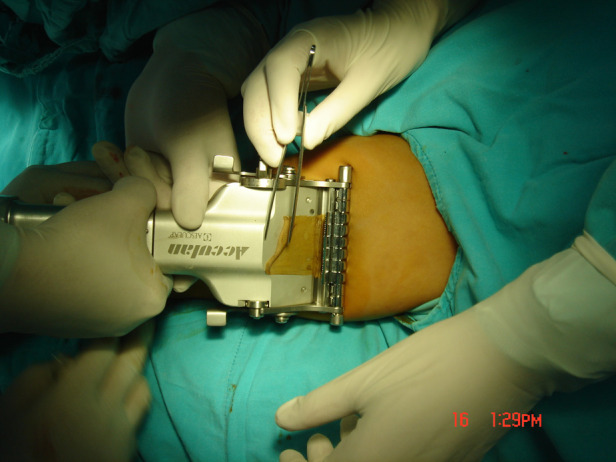
AESCULAP^®^ acculan 4.

STSG is more prone to wound contraction, hypoesthesia, hypopigmentation, or hyperpigmentation. There are two contractions of STSG, because the content of elastin fibers is low, and the contraction is relatively small in the first contraction, but in the second contraction, the contraction strength decreases with the thickness of the graft, so the area of STSG contraction is larger (74–76). And the thickness of STSG is proportional to the shrinking area (75). Due to the growth and development of adolescents, wound shrinkage will seriously affect the movement of the lower limbs, resulting in joint deformities. Contractions are currently mainly treated by physical methods, including functional exercises and splinting, and surgical release. Functional exercises are used by all surgeons, and splints are mostly used for contractions caused by burns, so appropriate physical therapy can reshape scar tissue to some extent (77). The acquisition of STSG requires the use of corresponding equipment, and the type of equipment depends on the size and thickness of the STSG. Before using a device that acquires STSGs, the number of STSGs needs to be determined in advance. STSGs in a small size range can be obtained directly using blades or scissors. STSGs with a slightly larger size range require a dermatome system. Among them, electric dermatome is the most common, including Brown dermatome, Zimmer dermatome, DavolSimon dermatome and Weck dermatome and other types of dermatome equipment, which can collect larger and more uniform STSG (39). A dial on the side of the dermatome can be turned to adjust the thickness of the graft (Figure 2). STSG can be divided into thin layers (0.005–0.012 in), medium layers (0.012–0.018 in), and thick layers (0.018–0.028 in). After obtaining the STSG, immerse it in sterile saline solution, and then manually divide the mesh according to the shape of the wound to adequately cover the wound.

The fixation of STSG is not as complicated as that of FTSG, and the blood supply between the wound bed and STSG is better than that of FTSG. The periphery of the graft is secured with common sutures or staples (78). There are generally two methods for securing STSG to the wound bed, including the basting suture technique and the helical suture technique (79, 80). Although STSG has a high survival rate, there is still a small chance of necrosis and even graft failure. Clinically, doctors have explored a variety of ways to improve the success rate. There are three main approaches to promoting STSG healing, including wound bed improvement, effective fixation of grafts, and other adjunctive physical therapy (81). Platelet-rich plasma (PRP) can improve wound bed conditions, thereby promoting STSG healing. Two studies in the treatment of complex skin defects and lower extremity ulcers have shown that PRP can promote STSG healing after uniformly receiving a combination of STSG and platelet-rich plasma (82, 83). Phenytoin ointment has a similar effect, and Younes et al. in a study of 16 patients with diabetic foot concluded that topical phenytoin ointment improved STSG survival (84). The secure fixation between the STSG and the wound bed prevents shear forces that interfere with graft healing. Both supportive dressings and Negative pressure wound therapy Negative pressure wound therapy(NPWT) are common means of effective graft fixation. It is worth adding that it is necessary to limit the patient's activities in the short term after STSG, which can reduce the generation of shear force. A short period of bed rest (2 days) reduces the incidence of wound infection, venous thrombosis, and does not affect graft survival (85, 86). Other adjuvant therapy, such as laser therapy, basic fibroblast growth factor (bFGF). It also has a certain promotion effect on the healing of STSG (87, 88).

### Epidermal graft

Epidermal grafting is an emerging technique to overcome scarring at the donor site. Epidermal transplantation (EG) is the transfer of an epidermal layer from the donor area to the wound bed. The epidermis is harvested by applying continuous negative pressure at the donor site to promote blister formation and transferred to the wound (89). Because the dermis is not involved, and the epidermis has a strong ability to regenerate and heal wounds quickly (renew every 4 weeks), the epidermis regenerates completely without scarring (38, 90). And EG, unlike the other two grafts, not only provides wound coverage but also stimulates wound regeneration (89). The pain-sensing nerves of the skin are located in the dermis, so EG is often painless, without the use of anesthesia, and is a day surgery. EG does not require entering the operating room and can be performed in an outpatient setting. Compared to FTSG and STSG, it is less invasive, easier to harvest, and has virtually no donor site morbidity (12, 91).

With the continuous development and improvement of the function and technology of the EG acquisition system, EG has been widely used in the outpatient setting. The first device developed by Kiistala in 1968 was the Dermovac, which consisted of a glass suction cup and a hand pump. Blister formation time is 1–2 h. Because of the long blister formation time and the large size of the device, it is not practical in practical work, so it has not been mass-produced (92, 93). Another common EG collection system is the syringe system. The principle of the device and the parts used are relatively simple. There are two syringes, one large and one small, and a three-way connector. Blister formation takes 1 h but requires surgical excision under anesthesia (94, 95). The newly developed CelluTome epidermis harvesting system consists of an automatic harvester, a vacuum head and a control unit. 30 min can produce EG more than 40cm^2^. Its entire procedure is fully automated, anesthesia-free and painless, and the graft size is consistent. Therefore, the device can easily provide EG in outpatient and community settings (96, 97). A study by Hachach-Haram et al. showed that after epidermal grafting of acute or chronic wounds in an outpatient setting with the device CelluTome, both the wound and the donor site healed well and the skin returned to its original appearance within 2 weeks (98, 99). Oliver J Smith and colleagues studied patient satisfaction with the graft in 20 patients who received FTSG and EG, respectively. Eighty percent of CelluTome patients are completely satisfied with the graft site results, and the cost of using the CelluTome system is much lower than that of patients with FSTG (96).

To further improve the success rate of EG, the wound environment can be improved, such as the formation of sufficiently fresh granulation tissue and infection control. It is also possible to sterilize the donor site with alcohol before heating or wetting it during the operation of EG, which can accelerate the formation of blisters. When transferring the graft, try to keep all the epidermis on top of the clear film dressing. Finally, the success rate of EG is improved by ensuring that the graft is in close contact with the wound (91). The specific mechanism of EG healing is currently unclear, but the following three mechanisms may play a role: keratinocyte activation, growth factor secretion, and re-epithelialization of the wound edge (33). Activated keratinocytes are so proliferative that small areas of EG can cover more than 30% of their total body surface area in burn patients (100, 101). And cytokines and extracellular matrix products secreted by keratinocytes play a key role in wound re-epithelialization (102). There are many types of growth factors, and one study found that EG contains vascular endothelial growth factor, TGF-α, platelet-derived growth factor AA, platelet-derived growth factor AB/BB, hepatocyte growth factor, and granulocyte colony-stimulating factor (100). Growth factors are known to accelerate wound healing and to stimulate the wound healing process (33). The re-epithelialization of the wound edge may be due to the graft-secreted growth factors at concentrations sufficient to modulate the gap junction proteins at the wound edge, thereby stimulating re-epithelialization at the wound edge (103). EG is mostly used for small, shallow wounds. Lower extremity avulsion injuries are often large-area, full-thickness skin defects, and epidermal transplantation alone is not effective. However, the combination of FTSG and STSG meshing treatments with epidermal grafts resulted in faster wound healing and increased wound coverage (104).

### Skin substitutes and wound dressings

The skin substitutes we mentioned here are composed of biomaterials. Others, such as xenografts, are not discussed here. The purpose of biomaterial skin substitutes is to provide extracellular matrix to accelerate wound healing (105). The primary role of the skin substitute is the same as the purpose of the graft, which is to prevent wound infection, keep the wound moist, and cover the defect. An absorbent dressing made of cotton wool was first developed in 1880 by Joseph Gamgee. Its advantages are also significant, such as being resistant to infection, durable, mass-produced, non-antigenic, and widely applicable (106). However, skin substitutes also have limitations, such as reduced vascularization, scarring, high price, and temporary wound coverage, ultimately requiring a secondary autologous skin graft (107, 108). The market for skin substitutes is huge, and countless researchers are devoted to the research and development of skin substitutes. There are many types of skin substitutes on the market. Artificial dermis has good biocompatibility, good adhesion, and easy access, avoiding the dilemma of insufficient autologous skin (38, 109). Moreover, it can also form a fresh wound on the exposed tendon, providing conditions for a second epidermal skin graft (53, 54).

The representative product of the double-dermis replacement, Integra has become one of the most popular double-dermis replacements since it was developed. It consists of a matrix of bovine tendon collagen type 1, shark chondroitin-6-sulfate, and silica gel (110). It enables long-term wound coverage, protects the wound from infection, and allows vascular invasion of the wound bed to degrade (111). For children with lower extremity avulsion injuries and multiple injuries throughout the body, the advantages of artificial dermis are significant. An example is the Integra Dermal Regeneration Template (IDRT), which has two layers. The outer layer is made of a silicone membrane and the inner layer consists of a matrix of cross-linked fibers. It can regenerate the functional dermis (112). Pelnac is also a two-layer dermal substitute consisting of a collagen matrix layer as the bottom layer and a semipermeable silicone layer as the outer layer. ZhenmuLv reported a case of a 48-year-old patient with a degloving injury of the upper extremity who was covered with Pelnac after initial surgical skin necrosis with satisfactory functional and aesthetic appearance. This is the first report of Pelnac addressing this complex skin defect (113). Lv et al. studied 16 patients with large skin defects of the lower extremities using the Pelnac dermal regeneration template and the recovery after secondary epidermal skin grafting, and 93.7% (15/16) of the patients achieved satisfactory functional outcomes. All patients were satisfied with the appearance. Attia and Tarek Elmenoufy treated 4 patients with extensive lower extremity avulsion injury using a combination of a NPWT and a dermal regeneration template (IDRT), and all 4 were healed with satisfactory results. The appearance of the reconstructed skin is near normal (15).

There are also emerging wound dressings that can also play a role in wound healing, such as Amnion is the thin translucent tissue in the innermost layer of the fetal membrane and is mainly used for canthus burns. It can be used as a temporary dressing over full-thickness or superficial burn wounds (114). Acticoat is a silver antibacterial dressing containing nanocrystalline silver that reduces healing time and reduces pain (115). It is suitable for full-thickness and partial-thickness wounds (116). Such as cell-laden hydrogels and hydrogel nanofiber mixtures, 3D printed skin substitutes, prevascularized skin equivalents, and even silk biomaterials as wound dressings use (Table 2). These emerging skin substitutes will have a huge impact in plastic surgery in the near future. However, the realization of these products still requires detailed and rigorous clinical studies to verify (117, 118).

**Table 2:** Different types of wound dressings.

Dressing	Material	Advantages	Applications	Products
Films and Membranes	CS/bioactive compounds CS/silver sulfadiazine/zeolite Cellulose/S Gel/CS/cinnamaldehyde	Good antioxidant activity proliferative effect adequate biocompatibility Antibacterial activity biodegradability	Minor split-thickness skin graft donor sites Secondary dressings for hydrogels, foams, alginates	Blisterfilm^TM^ (The Kendall Co) Carrafilm^TM^ (Carrington Laboratories)
Hydrogel	Keratin/glucose CS/liposomes containing curcumin	Good biocompatibility drug release behavior high swelling capacity	Non-exudative wounds Dry venous or arterial ulcers	Elasto-Gel^TM^ (SW Technologies) FlexiGel^TM^ (Smith / Nephew)
Hydrocolloid	Cross-linked polymer matrices	Provide mild cushioning Stimulate autolytic debridement	Partial-thickness burns Skin abrasions Superficial acute wounds	Tegasorb^TM^ (3 M) Hydrocol®II (UDL Laboratories)
Foam	Alg/Pec Polyurethane/propolis	Provide cell growth and adhesion Accelerate the wound healing proces	Wounds over bony prominences Mildly exudative wounds Donor sites	Biatain® (Coloplast) Biopatch® (Johnson / Johnson Medical) Flexzan® (UDL Laboratories)
Alginate	Seaweed Kelp-based polysaccharides	Highly absorbent	Bleeding wounds Donor sites Highly exudative wounds	Algisite^TM^ (Smith / Nephew) Algosteril® (Systagenix) KendallTM Curasorb^TM^ (Covidien) Kalginate® (DeRoyal)
Hydrofiber	Absorbent sheets Ribbons of sodium carboxymethylcellulose	Retain a moist environment Autolytic debridement	Partial-thickness donor sites Deep and exudative pressure ulcers Pyoderma gangrenosum, diabetic wounds	Aquacel® (ConvaTec)
Silver	Silver nitrate	Antibacterial activity	Superficially infected wounds	AQUACEL® Ag
Nanofiber	CS/PEO/Ag-ZnO NPs Alg-CS/Gtm Polycaprolactone/Gel/ amoxicillin/ZnO NPs	High antioxidant effect Antibacterial activity No cytotoxicity	Diabetic wounds	–
Sponges and Bandages	GO, chiitosan, hyaluronic acid	Excellent antibacterial activity Highly absorbent	Diabetic wounds	–

## Negative pressure wound therapy (NPWT)

NPWT is a new type of device that applies sub-atmospheric pressure to the surface of the wound dressing to drain fluid through a suction pump and collection device. Specifically, the open-cell foam that conforms to the shape of the wound is covered on the wound surface, and the foam collapses and shrinks under the action of negative pressure, which can prevent the wound skin edge from shrinking. In 1997, Argenta and Moryk developed vacuum assisted closure (VAC), which is widely used in acute and chronic wounds (119, 120). It uses negative pressure to remove necrotic tissue, change local blood supply, reduce edema, and promote local granulation tissue formation to accelerate wound healing and prevent wound infection (121–123). NPWT has two commonly used modes of instillation and dwell time-assisted NPWT (NPW Ti-d) and closed incision negative pressure therapy (ci NPT), both of which are used for 7 days (124). NPWT can be divided into three types, including black, white and gray. The black type has large pores and is mainly used to stimulate the growth of granulation tissue; the white type has small pores and mainly protects superficial wounds; the gray foam contains silver and is used for infected wounds (123).

Ordinary NPWT devices restrict activities, and with people's demands for quality of life and portability, portable and ultra-portable NPWTs have been developed. Theddeus O.H. et al. designed an EASEPort-NPWT. The main components of the device are simple and relatively inexpensive, including syringes, cans, tees, and barometers, which are all available in stores. However, the equipment is too rudimentary, and the effect on the treatment of exudate and the pressure transmission to the wound bed is not good, and further research is needed. At present, there are mainly two types of ultra-portable NPWTs: SnapNPWT and PICO. SnapNPWT uses a spring-loaded piston to deliver constant negative pressure to the disposable tank. It cannot select multiple pressure values and is not suitable for the need for high pressure to maintain hemodynamics and complex wounds with granulation growth (125). The PICO system is a single-use medical device, and the exudate is controlled by evaporation of the dressing, so no canister is required, which makes the pump ultra-light and portable. It can provide a variety of pressure values to choose from, and it can no longer be used after 7 days of continuous use, which will increase the financial burden of patients (126, 127).

NPWT can reduce wound inflammation indicators, a prospective cohort study involving 130 patients with soft tissue defects, they were randomly divided into NPWT group and routine dressing change (RDC) group. After 7 days, the granulation tissue coverage and bacterial clearance in the NPWT group were higher than those in the RDC group, and the levels of CRP, WBC and PCT in the NPWT group were lower than those in the RDC group. The results of this study show that NPWT has a significant effect after treatment and is more worthy of clinical application (121).

Numerous studies have shown that NPWT plays an integral role in the treatment of complex lower extremity wounds (128, 129). NPWT can be used to treat wound infections, open fractures, diabetic ulcers, skin grafts and other chronic wounds or wounds that are difficult to heal (6). G. Sakai et al. studied 12 patients whose skin flaps were degreased and sutured back in place after lower extremity avulsion injuries, of which 4 had necrosis of the degreased skin, and secondary skin grafting was performed on part of the wound. NPWT played a key role (129). JIAFUQU and colleagues used NPWT to treat 36 patients with a large soft tissue defect with exposed calf bone with a success rate of 80.56%, avoiding amputation and complicated surgery (130). NPWT is often difficult to fix in peripheral degloving injuries, and Tae Nagama reported the first case of using gauze packing and NPWT in the treatment of peripheral degloving injuries of the left lower extremity (131). Ranjit Chatterjee et al. proposed an upgraded version of the high suction capillary device of NPWT. Capillary Suction Devices (CSD) is made of reticulated foam with micron-scale openings (100–5 µm). Although the wound healing effect of CSD is basically the same as that of NPWT, it is economical and lightweight. It is light and does not affect the patient's activities, especially in the military (132). NPWT has many advantages, such as reducing the number of dressing changes, shortening the operation time and reducing the pain of patients, and the overall effect is significantly better than traditional dressing changes (2, 133, 134). However, NPWT is not suitable for all types of wounds, such as peripheral arterial disease of the lower extremity, uncontrolled wound infection, debridement of necrotic tissue, and tissue disruption caused by tumors (because NPWT stimulates angiogenesis and stimulates tumor growth) (123). NPWT also requires prompt dressing change because it can lead to overgrowth of granulation tissue and inhibit epithelialization (28). Of note, NPWT should be discontinued in the absence of any improvement between two consecutive dressing changes or after 1 week. Juveniles grow faster than adults and require more frequent dressing changes (135).

## Further cosmetic repair and follow-up

The ideal state of wound healing is scar-free healing (136, 137). In addition to EG, which can achieve the effect of scarless repair, other grafts often cause scars after wound healing due to the interference of various factors. Scarring of the skin may be the result of fibrosis during the three phases of wound healing (inflammation, new tissue formation, and remodeling) (138). The principle of action of existing drugs is to prevent fibrosis by early limiting neovascularization and inflammatory response (139–142). However, such drugs are often expensive and effective in the early stage, so the cost performance is not high. There are other treatment modalities, such as laser therapy, wearing compression stockings, steroids, etc. (77, 143, 144). Wound shrinkage is a normal physiological phenomenon that reduces the size of the skin defect to some extent (76, 145). However, in patients with extensive skin grafts, skin shrinkage can lead to deformity and limited joint mobility. And children have more severe wound shrinkage than adults (146). Treatment options to prevent wound shrinkage primarily counteract the mechanical forces generated by the shrinkage. If the effects of scarring and wound contraction continue to plague the patient's daily life during all of the above procedures, surgery may ultimately be used to improve quality of life. Due to the strong healing ability of children, most children can obtain satisfactory results in a short period of time. However, as children reach puberty, rapid growth and development may lead to functional deficits due to scarring (5). Therefore, frequent and intensive follow-up of the patient is required to assess the patient's range of motion, the impact of scarring on the patient, and recommend surgery if necessary. Even follow-up considers the patient's future occupation and whether multiple plastic surgeries will be required for optimal function and appearance (15). Therefore, long-term follow-up is very necessary (9).

## Conclusion

In conclusion, the management of juvenile lower extremity avulsion injuries is complicated, which is a challenge for surgeons. After debridement, anti-infection, and fluid replacement, the doctor should decide the type of skin graft according to the avulsion site, area, thickness of the avulsion tissue, and the survival of the avulsion tissue. FTSG is suitable for joints, STSG can cover most types of wounds, except joints. EG is not suitable for use alone in avulsion injuries and can be combined with FTSG or STSG to treat skin defects. Skin substitutes are particularly widely used, especially artificial dermis. The role of NPWT in the treatment of lower extremity avulsion injuries cannot be ignored. The appearance of scars after skin transplantation is inevitable. When scar contractures, hypertrophy, and limb function are affected, surgical treatment can be performed.
